# Cortico-basal ganglia networks subserving goal-directed behavior mediated by conditional visuo-goal association

**DOI:** 10.3389/fncir.2013.00158

**Published:** 2013-10-21

**Authors:** Eiji Hoshi

**Affiliations:** ^1^Frontal Lobe Function Project, Tokyo Metropolitan Institute of Medical ScienceTokyo, Japan; ^2^Japan Science and Technology Agency, Core Research for Evolutionary Science and TechnologyTokyo, Japan

**Keywords:** sensorimotor integration, visuomotor integration, goal, action, globus pallidus, executive function

## Abstract

Action is often executed according to information provided by a visual signal. As this type of behavior integrates two distinct neural representations, perception and action, it has been thought that identification of the neural mechanisms underlying this process will yield deeper insights into the principles underpinning goal-directed behavior. Based on a framework derived from conditional visuomotor association, prior studies have identified neural mechanisms in the dorsal premotor cortex (PMd), dorsolateral prefrontal cortex (dlPFC), ventrolateral prefrontal cortex (vlPFC), and basal ganglia (BG). However, applications resting solely on this conceptualization encounter problems related to generalization and flexibility, essential processes in executive function, because the association mode involves a direct one-to-one mapping of each visual signal onto a particular action. To overcome this problem, we extend this conceptualization and postulate a more general framework, conditional visuo-goal association. According to this new framework, the visual signal identifies an abstract behavioral goal, and an action is subsequently selected and executed to meet this goal. Neuronal activity recorded from the four key areas of the brains of monkeys performing a task involving conditional visuo-goal association revealed three major mechanisms underlying this process. First, visual-object signals are represented primarily in the vlPFC and BG. Second, all four areas are involved in initially determining the goals based on the visual signals, with the PMd and dlPFC playing major roles in maintaining the salience of the goals. Third, the cortical areas play major roles in specifying action, whereas the role of the BG in this process is restrictive. These new lines of evidence reveal that the four areas involved in conditional visuomotor association contribute to goal-directed behavior mediated by conditional visuo-goal association in an area-dependent manner.

## INTRODUCTION

When we drive a car and arrive at an intersection, we press the brake pedal if we see a red light, or we continue to press the gas pedal if we see a green light. More generally, we often act based on information provided by a visual signal. Because this type of goal-directed behavior integrates two forms of neural representations (i.e., perception and action), it is thought that identification of the neural mechanisms underlying their integration will yield insights into the fundamental principles underpinning goal-directed behavior. Some studies in this domain have used the framework provided by arbitrary visuomotor mapping (Passingham, 1993; Murray et al., 2000; Wise and Murray, 2000). In this paper, we will refer to this framework as conditional visuomotor association because it maintains that the integration of visual and motor signals is guided by behavioral rules (Wallis et al., 2001; Bunge et al., 2005) and because the association areas in the brain are believed to play a central role in this process (Goldman-Rakic, 1988; Miller and Cohen, 2001; Serrien et al., 2007; Fuster, 2008; Tanji and Hoshi, 2008; Passingham and Wise, 2012).

Accurate definitions of the goals and rules are therefore critical. Schall (2001) and Passingham and Wise (2012) presented clear definitions of the links between the goals and decisions and between actions and choices. Based on these studies, we here define the goals as “the objects or locations that an animal chooses as the target for its actions” (p. 71 in Passingham and Wise, 2012). In contrast, Bunge et al. (2005) and Bunge and Wallis (2008) defined the rules as ones that specify the most appropriate response under a given set of circumstances or contexts. In the case of goal-directed behavior, the rules are viewed as being implemented by individual neurons and/or neuronal networks for specifying the most appropriate goal or action under specific circumstances. Studies by White and Wise (1999) and Wallis et al. (2001) revealed that single neurons in the prefrontal cortex represent the rules, whereas studies by Hoshi et al. (2000) and Tanji and Hoshi (2001) suggested that the rules are implemented within networks or populations of neurons in the prefrontal cortex. Finally, Buschman et al. (2012) showed that the rules are implemented via oscillatory synchronization of ensembles of neurons. The multilevel representation of the rules is viewed as essential for cognitive control of goal-directed behavior (Miller, 2000).

**FIGURE 1:**
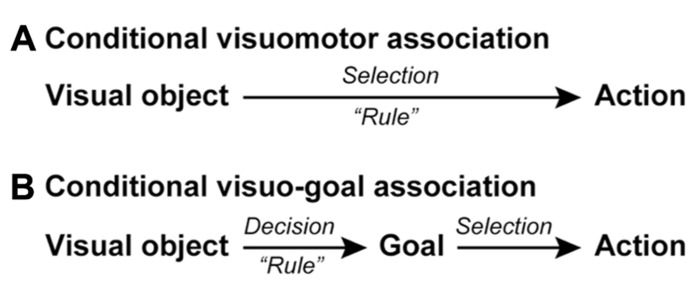
**Schematic representations of conditional visuomotor association and conditional visuo-goal association.** (A) In conditional visuomotor association, perceptual signals representing visual objects are directly mapped onto the motor signals (actions) in a *rule*-dependent manner to achieve an action selection. **(B)** In conditional visuo-goal association, perceptual signals are first mapped in a *rule*-dependent manner onto signals representing behavioral goals for making a goal decision. The goal-related signals are subsequently transformed into signals representing actions, corresponding to an action selection.

The conditional visuomotor association framework posits that neurons or networks directly link a visual signal to a bodily movement (action) in a rule-dependent manner (**Figure 1A**). However, this assumption encounters a problem when generalization and flexibility are required. Because the perceptual and action signals are supposed to be linked on a one-to-one basis, it is necessary to account for every combination of perceptual and action signals. In reality, this requirement is untenable. For example, responding to a red light involves squeezing a brake lever if one is riding a bicycle, pressing a brake pedal if one is driving a car, and stopping one’s movement if one is walking. Moreover, many varieties of visual signals and gestures can be the source of the instruction to stop. Actually, coming to a halt in the context of a signal to do so requires that numerous combinations of perceptual and action signals have been the foci of preparation. This requirement involving one-to-one combinations leads to another problem when the information provided by sensory signals changes or when a new motor response is required to execute the action implied by the information. For example, if a red light were to become the signal for proceeding or if the positions of the brake and gas pedals were reversed, we would need to relearn every combination of perceptual and action signals. Thus, neuronal networks that rely solely on computations based on conditional visuomotor associations would face major difficulties when information processing requires generalization or flexibility.

These flaws seem to rule out conditional visuomotor association as the mechanism underlying higher cognitive functions, which are characterized by flexibility and the ability to generalize (Milner, 1963; Luria, 1966). Thus, we must ask if we should discard this framework and seek a new conceptualization of the neural basis of information processing. Here, we would like to answer “no” and propose a new understanding of “goal” that renders the network responsible for conditional visuomotor association suitable as the underpinning of higher cognitive functions. Whereas the conditional visuomotor association framework assumes direct mapping between a visual signal and an actual movement (action), the new view is based on two additional assumptions (**Figure 1B**). First, it assumes that the visual signals provide information about an abstract behavioral goal instead of a concrete action. Second, it assumes that individuals subsequently specify or select an action to achieve that goal. We will refer to this new processing mode as conditional visuo-goal association because the visual signal is linked to a goal rather than to an action. The conditional visuo-goal association framework posits that neurons or networks directly link a visual signal to a behavioral goal in a rule-dependent manner (**Figure 1B**). This framework is considered to provide the goal-directed behavior with the generalization and flexibility. Once the goal is determined the subjects can specify or select an appropriate action to achieve the goal in various conditions, corresponding to the generalization. In addition, if the goal information provided by sensory signals changes, the subjects can address it by updating the association rules between the sensory signals and the goals, corresponding to the flexibility.

We will first review the mechanisms underlying conditional visuomotor association and then attempt to extend them to conditional visuo-goal association to elucidate how this network can serve as a basis of the higher cognitive functions that subserve goal-directed behavior.

## INVOLVEMENT OF THE DORSAL PREMOTOR CORTEX IN CONDITIONAL VISUOMOTOR ASSOCIATION

Pioneering studies by Halsband and Passingham (1982, 1985) and Petrides (1982, 1986) investigated the involvement of the premotor cortex (area 6) of monkeys in conditional visuomotor association. Halsband and Passingham (1982, 1985) trained monkeys to turn a handle when a yellow panel was presented and to pull a lever when a blue panel was presented. They found that monkeys with lesions of the bilateral premotor cortex but not of the bilateral frontal eye field failed to relearn the task in 1,000 trials. Petrides (1982) trained monkeys to grip a stick when a green circular bottle top was presented and to place their hand on a button when a blue and yellow toy truck was presented. He found that monkeys with lesions of the bilateral periarcuate areas, including the dorsal premotor cortex (PMd) and the frontal eye field, were severely impaired compared with normal monkeys or with monkeys with lesions around the bilateral principal sulci. Subsequently, Petrides (1986) showed that the periarcuate areas were involved in selecting between GO and NO-GO responses based on visual signals when GO and NO-GO were symmetrically rewarded. Importantly, these studies confirmed that monkeys with lesions centered on the premotor cortex were not impaired in perceiving visual signals or in executing movements. These observations revealed that the premotor cortex and the periarcuate areas are crucially involved in making conditional associations between visual signals and actions and in selecting between actions based on visual signals.

**FIGURE 2:**
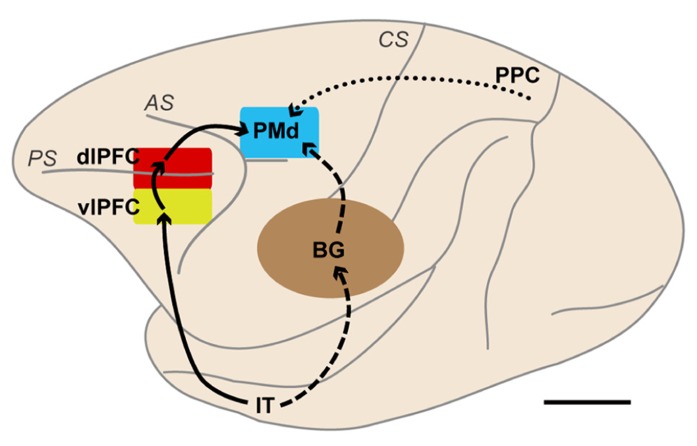
**Brain networks centered on the dorsal premotor cortex (PMd) involved in motor behavior based on visual object and visuospatial signals.** The solid lines indicate a pathway from the IT to the PMd via the vlPFC and dlPFC. The broken lines indicate pathways from the IT to the PMd. These two types of pathways are thought to be involved in behavior based on visual-object signals, such as those involved in conditional visuomotor association and conditional visuo-goal association. The dotted line indicates a pathway from the PPC to the PMd. This pathway is considered to carry visuospatial information. BG, basal ganglia; dlPFC, dorsolateral prefrontal cortex; IT, inferotemporal cortex; PMd, dorsal premotor cortex; PPC, posterior parietal cortex; vlPFC, ventrolateral prefrontal cortex; *AS*, arcuate sulcus; *CS*, central sulcus; *PS*, principal sulcus. Scale bar, 10 mm.

The lesions in these studies were fairly large and spanned multiple areas. In the studies conducted by Petrides (1982, 1986), lesions were made in both banks of the actuate sulcus, impairing the functions of both the frontal eye field and the premotor cortex. Although the lesions in the studies conducted by Halsband and Passingham (1982, 1985) were confined within the premotor cortex, they were made along both the superior and inferior limbs of the arcuate sulcus, leading to lesions of both the dorsal and ventral premotor cortices (Matelli et al., 1985; Rizzolatti and Luppino, 2001). Thus, the cortical areas that were most responsible remained elusive. To identify the responsible sites, Kurata and Hoffman (1994) injected the GABA_A_ receptor agonist muscimol to temporarily inactivate the PMd or the ventral premotor cortex (PMv). They first identified clusters of task-related neurons in the PMd and PMv while monkeys performed a conditional visuomotor association task that required them to perform a wrist flexion (extension) movement when a red (green) signal was presented. They next injected muscimol into the cluster in either the PMd or the PMv to reversibly inactivate it. They found that inactivation of the PMd cluster led to directional errors (i.e., impairments in selecting between the flexion and extension movements), whereas inactivation of the PMv cluster led to reduced movement amplitudes and velocities (i.e., impairments in movement execution). These findings provided compelling evidence that the PMd of monkeys is crucially involved in conditional visuomotor association (**Figure 2**).

Working with humans, Halsband and Freund (1990) revealed that patients with lesions that included the premotor cortex had difficulty selecting one of six arm movements according to visual signals, although the patients could execute the six different movements themselves and could perceive the sensory stimuli used as the instructions. Schluter et al. (1998) applied transcranial magnetic stimulation (TMS) to transiently interrupt local neural computations. They found that when TMS was applied over the PMd just after visual-cue presentation, which corresponds to the period of action selection, the selection process was delayed. Grafton et al. (1998) identified an activation focus of regional cerebral blood flow (rCBF) in the PMd while subjects chose between a power grip and a precision grip depending on the color of a LED. In a functional magnetic resonance imaging (fMRI) experiment, Amiez et al. (2006) determined that the PMd of humans was selectively activated when subjects selected one of four buttons in response to the presentation of one of four colors. These studies revealed that the PMd in humans is crucially involved in the selection of actions based on visual signals.

The PMd of human and non-human primates has been shown to play a crucial role in conditional visuomotor association (**Figure 2**). The specific aspects of information processing in which the PMd participates were revealed by recording neurons while monkeys performed a variety of motor tasks. Godschalk et al. (1981) found that PMd neurons responded to the presentation of visual signals and discharged in relation to the execution of reaching movements. Wise and colleagues recorded neurons while monkeys performed a variety of visuomotor tasks (Weinrich and Wise, 1982; Weinrich et al., 1984). They reported that PMd neurons strongly responded to the appearance of visuospatial signals and began to show sustained, set-related activity reflecting the direction of the forthcoming arm movements after the direction of forelimb movement was specified by visuospatial signals. Moreover, the set-related activity was more intense when a visuospatial information signaled execution of action than when it signaled inhibition of an action (Wise et al., 1983). When the motor plan was changed, the set-related activity also changed in accordance with the monkey’s motor intention (Wise and Mauritz, 1985). These observations revealed that the PMd is involved in the preparation and execution of movement based on visual signals.

Wise and colleagues subsequently conducted a series of landmark studies (Kurata and Wise, 1988; Mitz et al., 1991; Chen and Wise, 1995a, b). First, Kurata and Wise (1988) examined whether set-related activity was significantly modulated by type of visual signals. Subjects in their study participated in a conditional visuomotor association task, in which the color of the stimuli (conditional instructional stimuli) indirectly indicated the direction of an arm movement, and a directional task, in which the direction (left or right) of the visuospatial stimuli (directional instruction stimuli) directly indicated the direction of the movement. By examining the set-related activity of PMd neurons, they found that the activity of a great majority (81%) of neurons did not differ under the two task conditions. This observation indicates the relevance of set-related activity to the monkeys’ determination of the direction of a forelimb movement based on associated visual stimuli. Subsequently, Mitz et al. (1991) investigated the involvement of the PMd in learning conditional visuomotor association. They examined PMd neurons while monkeys learned new associations between visual images and the directions of handle movement and found that PMd neurons showed learning-dependent activity. Specifically, the visual, set-related, and movement-related activities associated with the same movement direction were more pronounced when the association was established than when it was not, indicating that PMd neurons are involved in the selection or retrieval of arm movements based on learned conditional associations between visual stimuli and movements as well as in the preparation and execution of movement, as discussed above. Chen and Wise (1995a) subsequently revealed that neurons in the supplementary eye field in the pre-PMd were involved in the conditional visuomotor associations for oculomotor behavior (Schlag and Schlag, 1987; Huerta and Kaas, 1990; Picard and Strick, 2001; Luppino et al., 2003). They identified learning-selective activity that was enhanced while monkeys learned new associations between visual signals and the direction of saccadic eye movements, and they specified the learning-dependent activity that was enhanced when such associations were established. They further found that a subset of neurons shows persistent differences in activity between novel and familiar information when performance is stable (learning-static effects; Chen and Wise, 1995b). These results revealed that the PMd and pre-PMd are involved in associating visual signals with actions with regard to arm and eye movements, respectively. From the perspectives of attention and intention, these observations suggest that the pre-PMd plays a major role in attentional or cognitive control of behavior with the prefrontal cortex, whereas the PMd plays a key role in the intentional control of actions or arm movements (Boussaoud and Wise, 1993a, b; di Pellegrino and Wise, 1993; Boussaoud, 2001; Lebedev and Wise, 2001; Rushworth et al., 2005; Abe and Hanakawa, 2009).

## INVOLVEMENT OF THE PMd IN PLANNING REACHING MOVEMENTS VIA CONDITIONAL VISUOMOTOR ASSOCIATION

**FIGURE 3:**
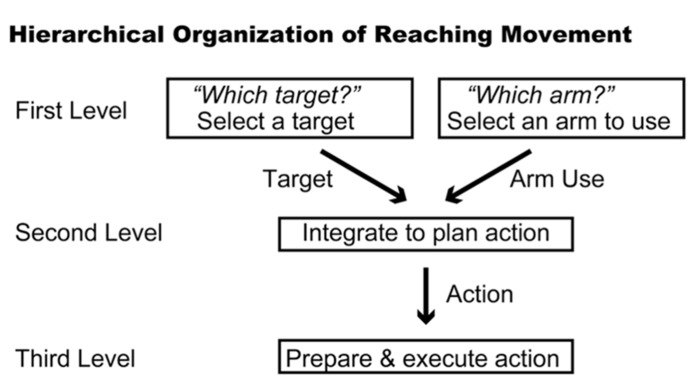
**Hierarchical organization of reaching movement.** Three levels of information processing are summarized schematically. The first level represents the components of the reaching movement, such as arm use and target location. The second level integrates these components to plan the reaching movement. The third level prepares and executes the planned movement.

The subjects in the studies discussed above could specify a forthcoming movement after the instruction cue was presented. However, prior studies also revealed that the PMd stores partial information about the direction or amplitude of movement when such information is provided in a stepwise manner (Riehle and Requin, 1989; Kurata, 1993). This phenomenon raises the intriguing possibility that the PMd may be involved in collecting and integrating diverse sets of information via the operation of conditional visuomotor association. In the case of planning a reaching movement, it is necessary to determine for which target to reach and which arm to use to do so. Thus, three hierarchical levels of information processing are presumably involved in the process of planning and executing a reaching movement (**Figure 3**). At the first level, information regarding which arm to use or for which target to reach is selected. At the second level, these two sets of information (the arm to be used and the location of the target) are collected and integrated to specify a reaching movement. This integration process must incorporate distinct types of information; although the arm is part of the participant’s body, the target exists outside of his or her body. After the reaching movement is planned, the neural processes at the third level prepare and execute it.

**FIGURE 4:**
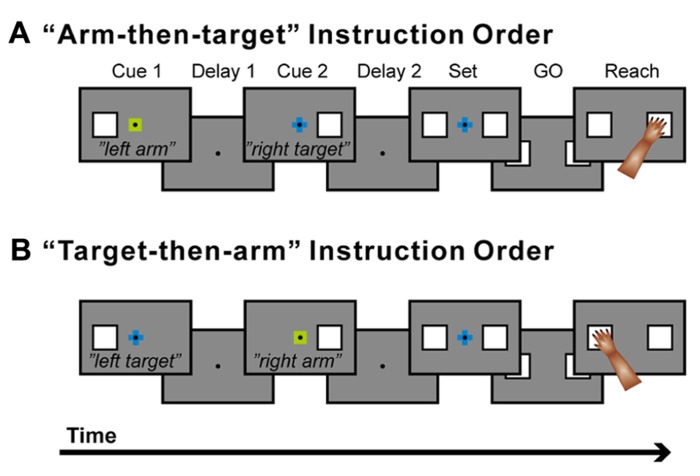
**Temporal sequence of behavioral events. (A)** The trial in which a signal about arm use (“arm”) was followed by a signal about the target to reach for (“target”). **(B)** The trial in which the two signal were given in the reverse order. When a monkey placed one hand on each touch pad and gazed at a fixation point (FP), the first instruction (cue 1; 400 ms in duration), which contained information about either the target location or which arm to use, was presented. A small, colored cue indicated the type of signal (i.e., whether it related to target location or arm use). A green square was used for an arm-use signal, whereas a blue cross was used for a target-location signal. At the same time, a white square appeared to the left or right of the FP and indicated laterality of arm use (for arm-related instructions) or target location (for target-related instructions). After the subsequent delay period (delay 1) that lasted ≥1,200 ms, the second instruction (cue 2: 400 ms) was given to complete the information required for the subsequent action. After the second delay (delay 2: ≥1,200 ms), squares appeared on each side of the fixation point (set cue: ≥1,000 ms), signaling the monkey to prepare to reach for the target when the fixation point disappeared (the GO signal). If the monkey subsequently reached for the appropriate target with the appropriate arm, s/he received a reward. The order of appearance of the target and arm signals was alternated in a block of 20 trials, and laterality was randomized within each block. A series of five 250 Hz tones was presented after a reward signaled a reversal of the order of the instructions.

A new behavioral task was developed to study the neuronal basis of these processes (Hoshi and Tanji, 2000). This task involves two sequential visual instruction cues separated by a delay (**Figures 4A,B**). One cue signals the location of the target (right or left), and the other cue signals which arm (right or left) to use. These instructions are given in the framework of what is known about conditional visuomotor association in that each compound visual signal is arbitrarily associated with each signal regarding arm use or target location. Therefore, after the first cue, it is necessary to collect and maintain information about the target location (if the first cue signals target location) or arm use (if the first cue signals arm use). After the second cue, monkeys were able to combine the two successive instructions about target location and arm use. Thereafter, the monkeys prepared to reach for the designated target with the designated arm, and they executed the reaching movement once a GO signal appeared (the disappearance of the fixation point). Altogether, the task design allowed us to study the neural mechanisms of the three levels of hierarchical organization underlying the reaching movement. It further allowed us to examine whether PMd neurons retrieve a partial motor instruction given by an arbitrarily associated visual signal.

**FIGURE 5:**
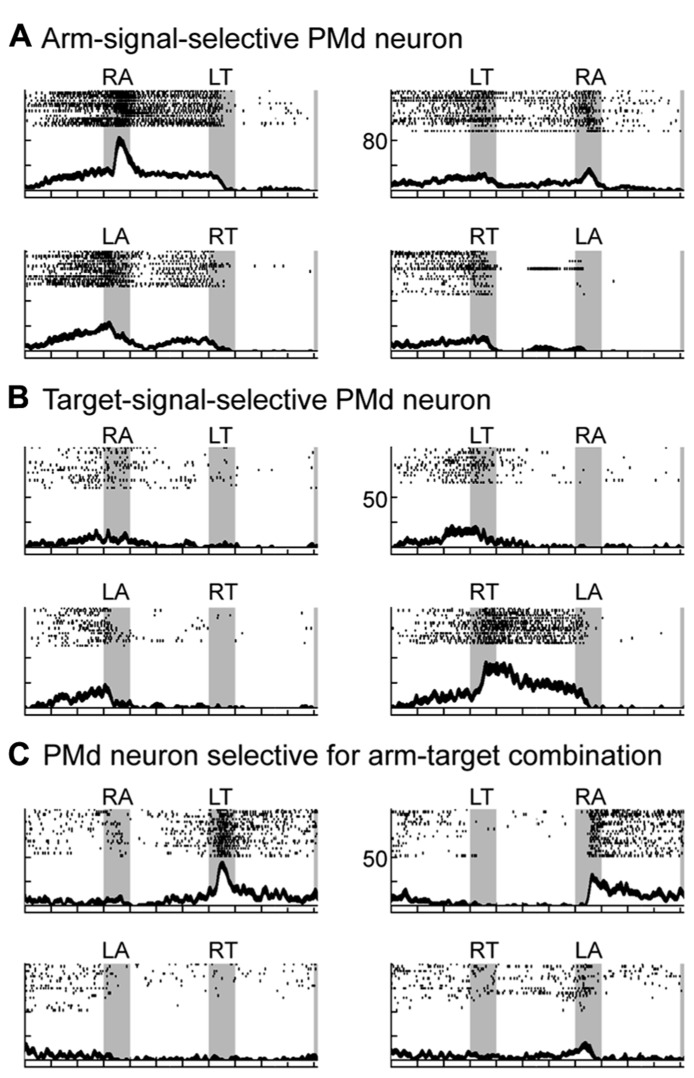
**Three types of neuronal activity in the PMd when monkeys planned a forthcoming reaching movement. (A–C)** Three examples of PMd neuronal activity presented with raster displays and plots of spike density functions (SDFs). Gray areas (from left to right) represent when the first, second, and set cues were presented. Tick marks on the abscissa are at 400-ms intervals. First and second signals are shown at the top of each panel (RA, right arm; LA, left arm; RT, right target; LT, left target). SDFs (Gaussian kernel, σ = 20 ms, mean ± SE) appear below each raster display. Raster plots and SDFs were aligned to the onset of the first and second signal and the onset of the set cue. The ordinate represents the instantaneous firing rate. Of the eight possible sequences of first and second cues (four instructions × two presentation orders), only four are illustrated. **(A)** Activity of this PMd neuron was greater when cue 1 signaled the use of the right arm (RA). **(B)** Activity of this PMd neuron was greater when cue 1 signaled the right target. **(C)** Activity of this PMd neuron was observed when the combination of the two signals was RA (use of the right arm) and LT (left target). Activity was similar, regardless of the order of the two instructions (adapted from Hoshi and Tanji, 2006).

By recording neurons in the PMd while the monkeys performed the task, three groups of neurons were identified that followed three distinct patterns of activity during the performance of this task (Hoshi and Tanji, 2000, 2006). Two patterns of neuronal activity were observed after the appearance of the first cue. The first group of neurons selectively responded to the appearance of the first cue about which arm to use, and the activity of this group persisted until the second cue was presented. For example, the neuron shown in **Figure 5A** discharged selectively after the appearance of the right-arm (RA) cue. The second group of neurons became active after the appearance of the cue regarding target location. The neuron shown in **Figure 5B** selectively discharged after the right-target (RT) cue was given, and, like those in the first group, its activity persisted until the second cue was presented. These findings revealed that PMd neurons retrieve and store a partial motor instruction, or a building block of action, when this information is embedded in a conditional visuomotor association. These processes correspond to the first level in the hierarchical organization of the reaching movement. When the second cue appeared, the third group of neurons became active. Neurons in this group seemed to represent the specific combination of the two instructions on arm use and target location given by the two cues. For example, the neuron shown in **Figure 5C** responded to the appearance of the second cue only when the combination of the two instructions on arm use and target location signaled the RA and the left target (LT). In other words, the third group of neurons was considered to contribute to the forthcoming reaching movement by integrating the two distinct sets of motor information, on arm use and target location. The existence of the three patterns of activity in the PMd suggests that this area contributes to planning reaching movements by collecting and integrating distinct sets of information on target location and arm use. These processes correspond to the second-level processing in the hierarchical organization and are the cardinal ones involved in action planning. We also found that during the preparation and execution periods of a reaching movement, PMd neurons selectively represented the specific combination of arm and target information (Hoshi and Tanji, 2002), which corresponded to the third level of processing in the hierarchical organization of the reaching movement. Altogether, the variety of activity found in the PMd suggests that this area is involved in all three levels of the processes underlying the generation of reaching movements. In humans, neurovascular activation in subjects performing a task with these demands indicated that the PMd represents the neural processes identified in monkeys (Beurze et al., 2007), revealing that the PMd of both human and non-human primates plays a crucial role in planning reaching movements.

In the behavioral task described above (Hoshi and Tanji, 2000), an identical instruction was presented with the first and second cues. An aim of this was to assess how each PM neuron responded to the first and second cues. By comparing the selectivity of the response of each neuron to each cue, we found that neurons selective for each instruction given by the first cue were evenly distributed among the three groups of the forthcoming action selectivity (arm use only, target location only, and both arm use and target location; see Figure 13A in Hoshi and Tanji, 2006). This suggests that there are no direct relationships between the selectivity after the first cue and that after the second cue. This indicates that PMd neurons conditionally represent the motor information in a planning-stage-dependent manner. This is consistent with a previous report showing that neuronal selectivity in the dorsomedial frontal cortex, which partly overlaps with the PMd, changed dynamically depending on the task requirements (Mann et al., 1988).

Taken together, the data discussed in this section suggest that the planning process of the PMd in humans and monkeys relies on conditional visuomotor association to retrieve the partial motor instructions provided by visual signals and integrate them for specific actions (Hoshi and Tanji, 2007).

## HOW IS THE PMD INVOLVED IN CONDITIONAL VISUOMOTOR ASSOCIATION?

Although these studies established that the PMd plays a central role in selecting or specifying an action and in representing and integrating the building blocks of action provided by arbitrarily associated visual signals, PMd neurons only rarely represented the visual-object signals themselves (Wallis and Miller, 2003). The absence of object-feature selectivity is consistent with the absence of direct connections with the inferotemporal cortex or the ventrolateral prefrontal cortex (vlPFC; Luppino et al., 2003), where visual features are amply represented (Ungerleider et al., 1982; Wilson et al., 1993; Tanaka, 1996; Orban, 2008). These observations lead to a question: How does the PMd contribute to conditional visuomotor association in the absence of its carrying information about the identity of visual objects?

Kalaska et al. (1998) proposed a theoretical account asserting that visual inputs are used in two different ways. First, the identity of a visual object provided through the ventral “what” visual pathway is used to make decisions about objectives and strategies for action. Second, the spatial visual signals provided through the dorsal “how” visual pathway are used to represent potential motor actions. They further proposed that the action to be executed is chosen through interaction between these two systems. This theoretical account suggests that the PMd may also contribute an abstract representation, such as “objectives and strategies for action,” to the process of conditional visuomotor association. Importantly, neurophysiological studies support this hypothesis. Specifically, Cisek and Kalaska (2002, 2005) developed a task in which two potential targets (red and blue) were initially presented, and monkeys chose between them based on the target color. Their findings revealed that PMd neurons initially represent potential reach directions and subsequently represent the direction of the selected reach target. Based on these findings, they proposed that multiple reach options are initially specified and then gradually eliminated in competition for which is to be actually executed. Subsequently, Cisek and Kalaska (2004) showed that PMd neurons carry task-relevant signals when monkeys observe a learned, visuomotor task being performed by others as well as when monkeys perform the task themselves. Bastian et al. (2003) reported that the activity of PMd neurons is modulated by the degree of certainty that the selected object is indeed the correct reach target. Wallis and Miller (2003) found that PMd neurons can represent abstract information that is not directly related to the movement parameters. In that study, monkeys were required to apply a “same” or “different” rule to execute or withhold action in response to two successively presented pictures. They found that neurons in both the PMd and prefrontal cortex represented abstract rules, which are more strongly represented in the PMd than in the prefrontal cortex. In oculomotor behavior, Olson and colleagues revealed that neurons in the supplementary eye field in the pre-PMd represented the relative position of two target objects for saccadic eye movements (Olson and Tremblay, 2000; Tremblay et al., 2002). These crucial observations indicate that PMd neurons reflect abstract representation that is not directly related to the movement in question in advance of the specification of an action.

## INVOLVEMENT OF THE PMd IN CONDITIONAL VISUO-GOAL ASSOCIATION

**FIGURE 6:**
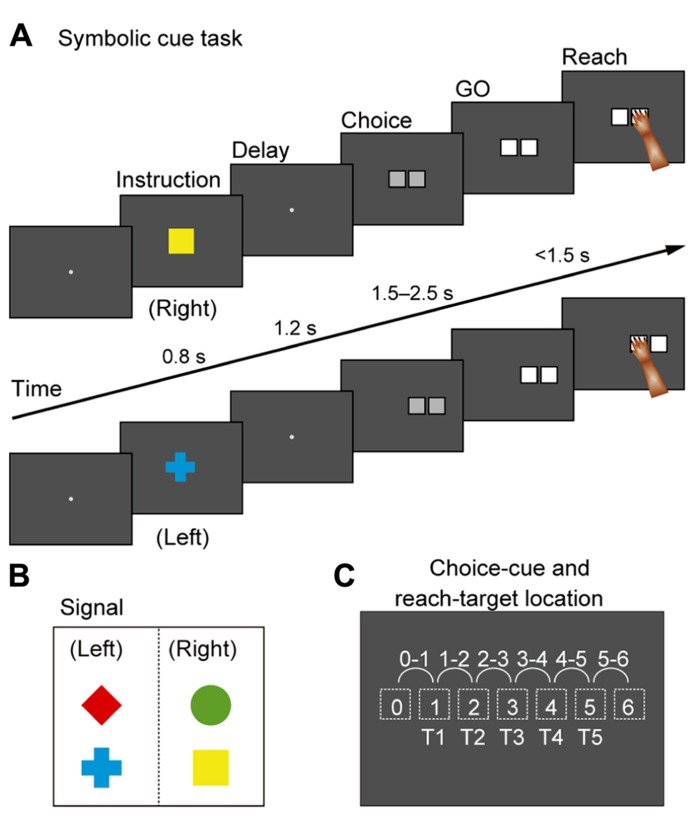
**Symbolic cue task. (A)** Temporal sequence of behavioral events in the symbolic cue task. If the monkey continued to gaze at the fixation point for 1,200 ms, a cue was randomly presented for 800 ms to signal the animal to select either the right or the left target (i.e., the behavioral goal). A green circle and a yellow square signaled selection of the target on the right, whereas a red diamond and a blue cross indicated that the left target should be selected **(B)**. Because no information about future targets was available at this stage, the monkeys were required to select right or left without specifying a forthcoming action. If the monkey continued to gaze at the fixation point for 1,200 ms during the subsequent delay, a choice cue consisting of two gray squares appeared at one of six different locations on the screen **(C)**. At this point, the animal could specify what to do (i.e., action) for the first time. After 1,500–2,500 ms, the color changed from gray to white (the GO signal). If monkeys reached for the target with their right arm, they received a fruit juice reward 500 ms after touching the correct square. **(B)** Visual signals used to designate selection of left or right in the forthcoming choice cue. **(C)** Locations of the choice cue and target on the screen. For the choice cue, two gray squares appeared at neighboring positions (locations 0–6, depicted with dotted squares). The target position was selected from five potential targets (T1–T5) that were located on the left or the right of the choice cue.

Based on this account, we developed a new behavioral task for monkeys (Nakayama et al., 2008) that includes an abstract representation of behavior; a cue evoking this abstract representation was inserted between a visual object and an action (**Figure 1B**). This design was also based on the notion that a visual signal often indicates an abstract aspect of behavior rather than an actual movement. For instance, a red traffic light instructs us to “stop”; subsequently, we execute an action to “stop” (e.g., squeezing a bicycle brake lever or pressing a car brake pedal). Thus, it can be seen that we first make a decision about a behavioral goal (“stop”) based on a sensory signal (a red traffic light) and subsequently choose the appropriate action to achieve the goal. **Figure 6** shows the time sequence of the behavioral task (the symbolic cue task; Nakayama et al., 2008). This task had the following three behavioral phases, which were temporally separated: (1) determining the behavioral goal on the basis of the visual-object cue; (2) specifying or selecting an action based on the information about the behavioral goal and the spatial position of the choice cue; (3) preparing and executing the action. The visual object indicated that either the LT or the RT should be selected later in the task period, but it did not indicate the exact position of the future target. During this phase, the monkeys could determine only the relative position of the reach target (an abstract behavioral goal), but no specific information about the actual reach target was available because the choice cue, consisting of two potential targets, was presented later at various positions on the screen. At this stage, the monkeys could determine, for the first time, where to reach on the screen (an action) by transforming the behavioral goal into an action based on the choice-cue position. After a delay, the color changed from gray to white, which served as the GO signal. In this task, “the relative position of the reach target” corresponds to “the locations that an animal choses as the targets for its actions” (i.e., the goals), but not “the representations specifying which goal is appropriate in a given context” (i.e., the rules; see Introduction for the definitions of goals and rules). Thus, by analyzing the activity of neurons while monkeys performed the task, we were able to examine the information-processing operation from the perception of visual objects to the specification of the action mediated by the abstract behavioral goal.

**FIGURE 7:**
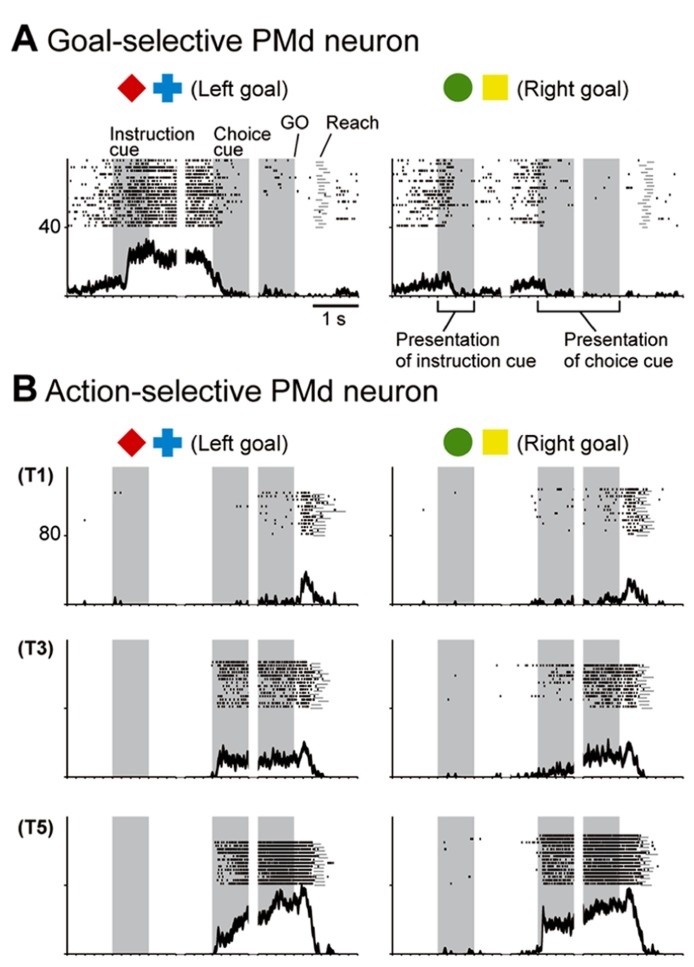
**Two examples of neurons in the PMd. (A)** Goal-related activity of a PMd neuron. Activity of this PMd neuron increased when either a red diamond or a blue cross was used to specify the left target. **(B)** Action-related activity of a PMd neuron. After choice-cue onset, this PMd neuron exhibited more activity when the correct target was located on the right side of the screen (T5), regardless of the goal. Of the five positions (T1–T5), only three (T1, T3, and T5) are displayed here. **(A,B)** Rasters and spike-density functions (smoothed using a Gaussian kernel; σ = 10 ms, mean ± SEM) indicate activity in sorted trials. The ordinate represents the instantaneous firing rate (spikes/s). Neuronal activity was aligned with the onset of the instruction, choice-cue, and GO signals. Gray areas on the left indicate when the instruction was presented, and gray areas in the middle and on the right represent when the choice cue was presented. Tick marks on the horizontal axis are placed at 200-ms intervals (adapted from Nakayama et al., 2008).

**FIGURE 8:**
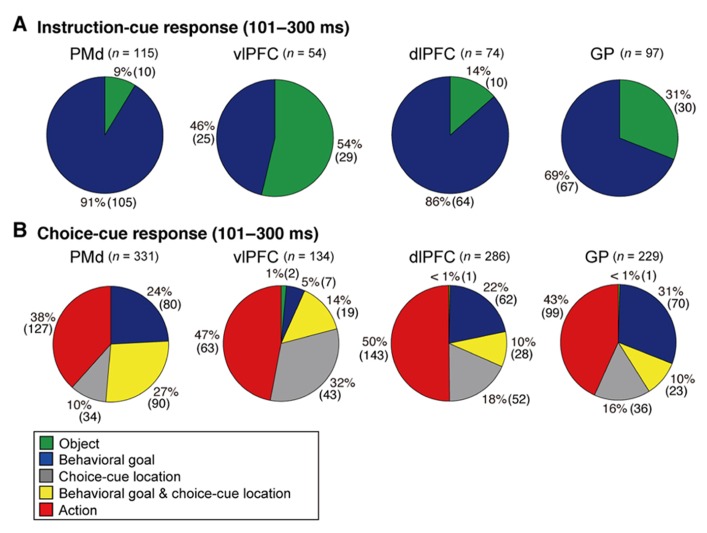
**Distribution of selective neurons in the PMd, vlPFC, dlPFC, and GP.** Pie charts summarize the proportion of neurons classified into five categories. Two sets of data are shown for 101–300 ms after the onset of instruction cue **(A)** and 101–300 ms after the onset of the choice cue **(B)**. Each category is color coded according to the inset. The parentheses enclose the number of neurons. Green, object neurons; Blue, goal neurons; Gray, neurons selective for choice-cue location; Yellow, neurons selective for both goal and choice-cue location; Red, action neurons (adapted from Arimura et al., 2013).

While monkeys performed this task, we first recorded neurons from the PMd. The activity of PMd neurons initially reflected the behavioral goal, reaching toward the LT or the RT after the choice cue signaled by the visual objects (**Figure 7A**), although it was rarely selective for the visual objects themselves (**Figure 8A**). Subsequently, when a pair of potential targets was presented as the choice cue, information about the spatial position of the choice cue was rapidly combined with information about the behavioral goal (**Figure 8B**), resulting in the development of an action representation (**Figure 7B**), which eventually replaced the behavioral goal representation. Our observations also revealed a subset of PMd neurons that first exhibited activity representing the behavioral goal, which changed into activity representing a mixture of the behavioral goal and the choice-cue location after the appearance of the choice cue, suggesting that these neurons directly contributed to the transition between the goal-related and the action-related use of the information. These results suggest that the PMd hosts a neural network involved in integrating the behavioral goals retrieved from visual-object signals with the locations of choice cues to specify forthcoming actions.

As discussed above, prior studies have indicated that the PMd employs abstract representations as a part of an information-processing operation involving partial motor instructions, the rule for linking visual-signal processing to action, the potential reach direction, others’ performance of a learned visuomotor task, and the certainty with which a target is selected. Our study revealed that the PMd represents abstract behavioral goals derived from arbitrarily associated visual signals that specify later action. These results provide compelling evidence that the PMd is involved not only in the preparation and execution of action but also in the representation of the abstract information needed to specify an action. In general, the PMd is involved not only in visuomotor association but also in conditional visuo-goal association, which includes an abstract representation of a behavioral goal as a core element. Consistent with this, Hanakawa et al. (2002) showed that the PMd in humans is active during mental-operation tasks, such as mental calculation, that do not involve any immediate overt movement. Based on this finding, they proposed that the PMd plays a major role in motor behavior requiring cognitive manipulation of abstract representations. The goal neurons found by Nakayama et al. (2008) were considered to play an important role in this process; the goal is the abstract representation that does not directly relate to action execution, but goal representation is crucial for specifying the action.

## SOURCES OF PMd INFORMATION ABOUT ABSTRACT GOALS

Nakayama et al. (2008) reported that the PMd retrieves the abstract information derived from a visual-object signal even though it rarely represents that information. This paradox raises an intriguing question: From which areas does the PMd receive such abstract information? To gain insight into this issue, the temporal development of the selection of the behavioral goal was compared with the development of visuospatial selectivity (Yamagata et al., 2009). PMd neurons represented the initial visuospatial signals 90 ms after the presentation of visual stimuli. The rapidity of this process suggests that the PMd receives this signal from the directly interconnected posterior parietal cortex, where visuospatial signals are amply represented (**Figure 2**; Johnson et al., 1993; Galletti et al., 1997; Snyder et al., 1997; Wise et al., 1997; Matelli et al., 1998; Colby and Goldberg, 1999; Pesaran et al., 2008). By contrast, the development of the goal representation was found to take much more time; PMd neurons began to represent the goals 150 ms after the visual object was presented. This 60-ms delay indicates that goal signals reach the PMd via distinct pathways. Based on the following findings, we hypothesized that the basal ganglia (BG) and/or lateral prefrontal cortex mediate these pathways.

The BG and lateral prefrontal cortex play crucial roles in associating visual signals with actions in a goal-oriented and adaptive manner (Graybiel et al., 1994; Wise et al., 1996; Konishi et al., 1998; Rainer et al., 1998; Kim and Shadlen, 1999; Hollerman et al., 2000; Everling et al., 2002; Nieder et al., 2002; Packard and Knowlton, 2002; Takeda and Funahashi, 2002; Barraclough et al., 2004; Genovesio et al., 2005; Saito et al., 2005; Sakagami and Pan, 2007; Sakai, 2008; Buckley et al., 2009; Hussar and Pasternak, 2009; Yoshida and Tanaka, 2009; Cisek and Kalaska, 2010; Goodwin et al., 2012; Meyers et al., 2012; Swaminathan and Freedman, 2012). Substantial structural interactions between the BG and the frontal cortex are considered to provide the structural basis for this process (Alexander et al., 1986; Flaherty and Graybiel, 1994; Inase and Tanji, 1994; Middleton and Strick, 2000; Nambu et al., 2002; Graybiel, 2008). Neurovascular activation in humans performing conditional visuomotor association was observed in the vlPFC and the BG as well as in the PMd (Toni et al., 2001, 2002). Because the BG and vlPFC receive inputs from the inferotemporal cortex, where visual-object signals are amply represented (Saint-Cyr et al., 1990; Webster et al., 1993, 1994; Middleton and Strick, 1994; Schall et al., 1995; Cheng et al., 1997; Petrides and Pandya, 2002), these projections are thought to provide visual-object signals to these areas. Lesion studies of monkeys have revealed that impairments in conditional visuomotor association arise from disruptions in the vlPFC (Wang et al., 2000; Bussey et al., 2001), the interconnection between the vlPFC and the inferotemporal cortex (Eacott and Gaffan, 1992; Bussey et al., 2002), and the interaction between the BG and the PMd (Nixon et al., 2004). From a functional perspective, vlPFC neurons have been shown to integrate the two sets of information about object features and the selected directions of saccades (Asaad et al., 1998). Furthermore, association learning in the BG (the striatum) has been shown to precede that in the lateral PFC (Pasupathy and Miller, 2005). Modulation of the activity of neurons in the globus pallidus (GP) is enhanced when the stimulus–response association is familiar (Inase et al., 2001). Similarly, the activity of neurons in the striatum is enhanced during learning of visuomotor associations (Hadj-Bouziane and Boussaoud, 2003). Moreover, the learning of associations between visual objects and movements has been shown to progress simultaneously in striatal and PMd neurons (Brasted and Wise, 2004). These observations suggest that the BG and vlPFC are crucially involved in conditional visuomotor association and that the interaction between the PMd and these areas is essential to the successful operation of this process.

However, because the PMd does not receive direct inputs from either area (Barbas and Pandya, 1987; Webster et al., 1994; Luppino et al., 2003), this anatomical connection remains to be proven. To address this issue, the rabies virus was transneuronally traced in macaque monkeys to provide evidence for communication across synapses between the PMd and the vlPFC and BG (Takahara et al., 2012). The rabies virus is transported across synapses from the postsynaptic to presynaptic neurons in a time-dependent manner. This feature allowed the identification of the areas that project across synapses to the PMd after injection of the rabies virus into the PMd.

Initially, the corticocortical pathways from the vlPFC to the PMd were analyzed. Fast Blue (a conventional retrograde tracer) was injected into the PMd to identify the cortical areas that send projection fibers directly to the PMd. Considerable retrograde labeling occurred in the dlPFC, area F7 (pre-PMd), pre-supplementary motor area (pre-SMA), and PMv (Barbas and Pandya, 1987; Lu et al., 1994; Luppino et al., 2003), whereas the vlPFC was virtually devoid of neuronal labeling. Subsequently, the rabies virus was injected into the PMd. Three days after the rabies injections, second-order neurons were newly labeled in the vlPFC, providing evidence that the vlPFC sends disynaptic projections to the PMd. To identify the areas that mediate the pathways from the vlPFC to the PMd, an anterograde/retrograde dual-labeling experiment was conducted in individual monkeys. By examining the distribution of axon terminals labeled from the vlPFC and cell bodies labeled from the PMd, substantial overlap was found in the dlPFC (area 46d), area F7 (pre-PMd), and pre-SMA (**Figure 9**). These results indicate that vlPFC outflow is directed toward the PMd in a multisynaptic fashion through these areas (**Figure 2**).

**FIGURE 9:**
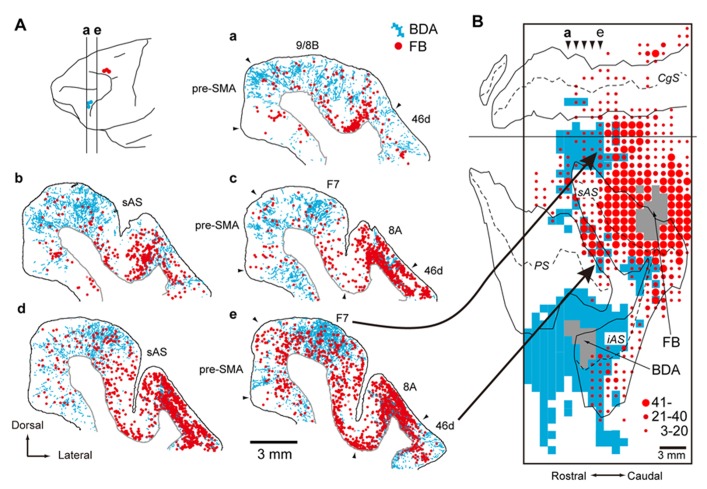
**Overlaps of axon terminals arising from the vlPFC and cell bodies projecting to the PMd in the frontal cortex. (A)** Five representative coronal sections are arranged rostrocaudally. The approximate rostrocaudal levels of the sections (a–e) are indicated in the lateral view of the brain (with the sites of BDA and FB injections specified by blue and red circles, respectively). Labels in blue represent axon terminals labeled with BDA injected into vlPFC (area 45), and labels in red represent cell bodies labeled with FB injected into PMd. **(B)** A two-dimensional density map showing the distribution patterns of axon terminals labeled with BDA and cell bodies labeled with FB. The bins (900 × 1,000 μm) where the BDA-labeled axon terminals were observed appear in blue. The box encloses the area where BDA terminals were investigated. The gray zone denotes the extent of the FB or BDA injection sites. Three different sizes of filled circles represent the numbers of neurons labeled with FB. Arrowheads (a–e) point to the approximate rostrocaudal levels of coronal sections (a–e) shown in **(A)**. BDA, biotinylated dextran amine; FB, Fast Blue. AS, arcuate sulcus; CgS, cingulate sulcus; F7, area F7 (Luppino et al., 2003); iAS, inferior limb of AS; PS, principal sulcus; sAS, superior limb of AS (adapted from Takahara et al., 2012).

Subsequently, the multisynaptic projections from the BG to the PMd were analyzed (Saga et al., 2011) after the injection of the rabies virus into the PMd. Specifically, second-order neurons were identified in the internal segment of the globus pallidus (GPi) and the substantia nigra pars reticulata (SNr). Labeled GPi neurons were found in the dorsal portion at the rostrocaudal middle level and in the caudoventral portion. In the SNr, labeled neurons were widespread in the rostrocaudal direction. Subsequently, third-order neuron labeling was observed in the external segment of the globus pallidus (GPe), the subthalamic nucleus (STN), and the striatum. In the GPe, the labeled neurons were observed over a broad territory centered in the rostral and dorsal portions. In the STN, PMd injection resulted in extensive labeling over the nucleus, especially in the dorsoventral middle and dorsal portions. In the striatum, labeled neurons were widespread in the striatal cell bridge region and neighboring areas, as well as in the ventral striatum. These results provide evidence that the PMd receives substantial inputs across synapses from the BG. Taken together with prior studies revealing the projections from the PMd to the striatum and the STN (Nambu et al., 1997; Takada et al., 1998; Tachibana et al., 2004), it appears that the PMd and BG form loop circuits that subserve multiple aspects of information processing (Alexander et al., 1986; Alexander and Crutcher, 1990a).

These anatomical studies revealed that the PMd receives inputs across synapses from the vlPFC and the BG (**Figure 2**), raising the intriguing possibility that the circuits linking the PMd to the vlPFC and/or the BG may be involved in retrieving the abstract goals from the visual-object signals. To test this hypothesis, the response properties of neurons in the lateral PFC and the BG were compared with those of neurons in the PMd.

## INVOLVEMENT OF THE PREFRONTAL CORTEX IN CONDITIONAL VISUO-GOAL ASSOCIATION

These anatomical studies suggest that the PMd receives inputs from the vlPFC partly via the dlPFC, which has been implicated in behavioral planning (Luria, 1966; Shallice, 1982; Funahashi et al., 1989, 1993; Frith et al., 1991; Goel and Grafman, 1995; Rowe et al., 2000; Averbeck et al., 2002, 2003; Hoshi and Tanji, 2004a; Mushiake et al., 2006; Mansouri et al., 2007). Based on these observations, the neuronal activity in the vlPFC and dlPFC was examined while monkeys performed the symbolic cue task involving conditional visuo-goal association (Yamagata et al., 2012).

**FIGURE 10:**
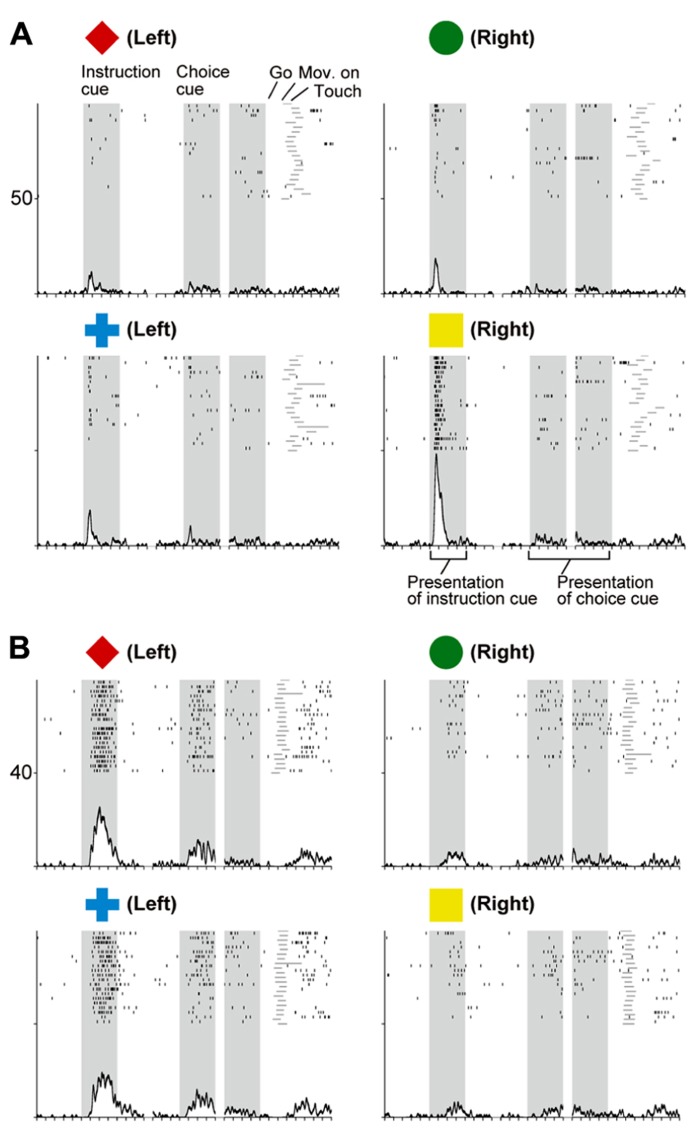
**Two examples of neurons in the prefrontal cortex selective for visual objects (A) and behavioral goals (B). (A)** Activity of this vlPFC neuron increased when the yellow square was presented. **(B)** Activity of this dlPFC neuron increased when the instruction was presented and either a red diamond or a blue cross was used to specify the left target. The display formats are the same as those used in **Figure 7** (adapted from Yamagata et al., 2012).

When the instruction cue was presented, a sizeable number of vlPFC neurons exhibited responses that were selective for visual-object features (**Figure 8A**). For example, the neuron shown in **Figure 10A** strongly responded to the appearance of a yellow square. This object representation is consistent with anatomical reports that the vlPFC receives input from the inferotemporal cortex and with prior studies reporting ample object representations by vlPFC neurons (Wilson et al., 1993; O Scalaidhe et al., 1997, 1999). The existence of object-selective activity suggests that vlPFC neurons participate substantially in encoding visual-object features for subsequent use. We found that the object-feature selectivity in the vlPFC was rapidly replaced with activity that was selective for behavioral goals. In contrast, dlPFC neurons rarely represented visual-object features; instead, they began to represent goals after the instruction cue was presented (**Figure 8A**). For example, the dlPFC neuron shown in **Figure 10B** selectively responded to the appearance of a red diamond and a blue cross signaling the LT. The limited representation of the visual-object signals in the dlPFC is in accord with the paucity of anatomical connectivity between the dlPFC and the inferotemporal cortex (Petrides and Pandya, 1999). These observations indicate that both the vlPFC and dlPFC are involved in retrieving the goals signaled by visual objects. However, the two areas are involved in different ways: the visual-object feature was represented in the vlPFC when the neural representations of the goal developed, whereas the goal representation in the dlPFC developed independently of any encoding of object features. From a perspective of a categorization, Freedman et al. (2001) revealed that lateral PFC neurons categorize visual stimuli as “cats” and “dogs,” whereas the observations made by Nakayama et al. (2008) and Yamagata et al. (2012) suggest that lateral PFC and PMd neurons categorize visual stimuli as associated with right and left behavioral goals.

**FIGURE 11:**
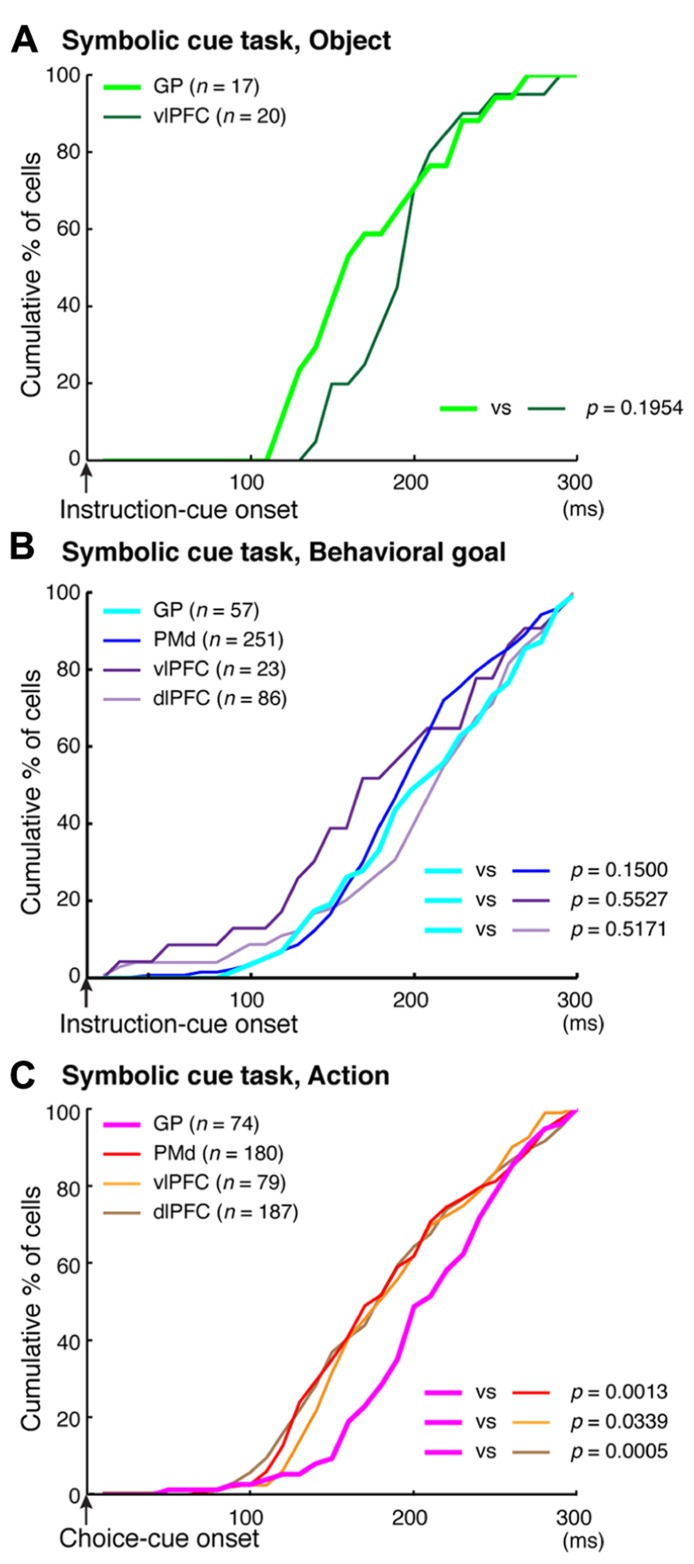
**Comparison of the development of selectivity for visual objects, behavioral goals, and actions. (A)** Cumulative fractions of selectivity onset for visual objects in the GP (light green) and vlPFC (dark green) after instruction-cue onset in the symbolic cue task. **(B)** Cumulative fractions of selectivity onset for the behavioral goal in the GP (light blue), PMd (dark blue), vlPFC (dark purple), and dlPFC (light purple) after instruction-cue presentation in the symbolic cue task. **(C)** Cumulative fractions of the onset of action selectivity in the GP (pink), PMd (red), vlPFC (orange), and dlPFC (brown) after choice-cue onset in the symbolic cue task. **(A–C)** The *p*-values indicate the results of the statistical analysis (Kolmogorov–Smirnov test) between the GP and the other three areas (adapted from Arimura et al., 2013).

To better understand the flow of information across the vlPFC, dlPFC, and PMd, the timing of the emergence of selectivity was compared with a measure of population selectivity (Yamagata et al., 2012). In the vlPFC, object selectivity began 130 ms after onset of the instruction cue, whereas goal selectivity began 150 ms after that point, indicating that goal selectivity developed in the vlPFC while object information was represented. In the dlPFC, goal selectivity developed 170 ms after the instruction-cue onset. Based on these findings, we propose the following hypothesis regarding the involvement of the vlPFC and dlPFC in conditional visuo-goal association: Neurons in the vlPFC retrieve goal signals (150 ms after instruction-cue onset) from the visual-object signals that are already represented there (130 ms). Then, the retrieved signals are transferred via cortico-cortical connections to the dlPFC, where they trigger the goal representation (170 ms). If the development of the goal representation in the PMd were later than that in the dlPFC, we could propose the operation of a cortico-cortical pathway from the vlPFC to the PMd via the dlPFC. However, goal representation developed in the PMd 150 ms after the onset of the instruction cue, which was comparable to the timing in the vlPFC (150 ms) and earlier than that in the dlPFC (170 ms). Furthermore, the selectivity developed significantly earlier in the PMd than in the dlPFC for individual neurons representing the behavioral goals (**Figure 11B**, Kolmogorov–Smirnov test, *p *= 0.0293). These observations reveal that goal representation develops almost simultaneously in the PMd and vlPFC, which are indirectly interconnected, whereas goal development in the dlPFC, which is thought to mediate the pathway between these areas, tends to follow that in the PMd and vlPFC. These data did not support the view that the goal signals generated in the vlPFC travel cortico-cortically to the PMd via the dlPFC. Wallis and Miller (2003) showed this kind of non-hierarchal representation between the PFC and the PMd in the representation of an abstract, matching-to-sample, or a non-matching-to-sample rule related to initiating action and revealed that PMd neurons begin to encode the rule information earlier than PFC neurons do.

## INVOLVEMENT OF THE CORTICO-BG CIRCUITS IN CONDITIONAL VISUO-GOAL ASSOCIATION

**FIGURE 12:**
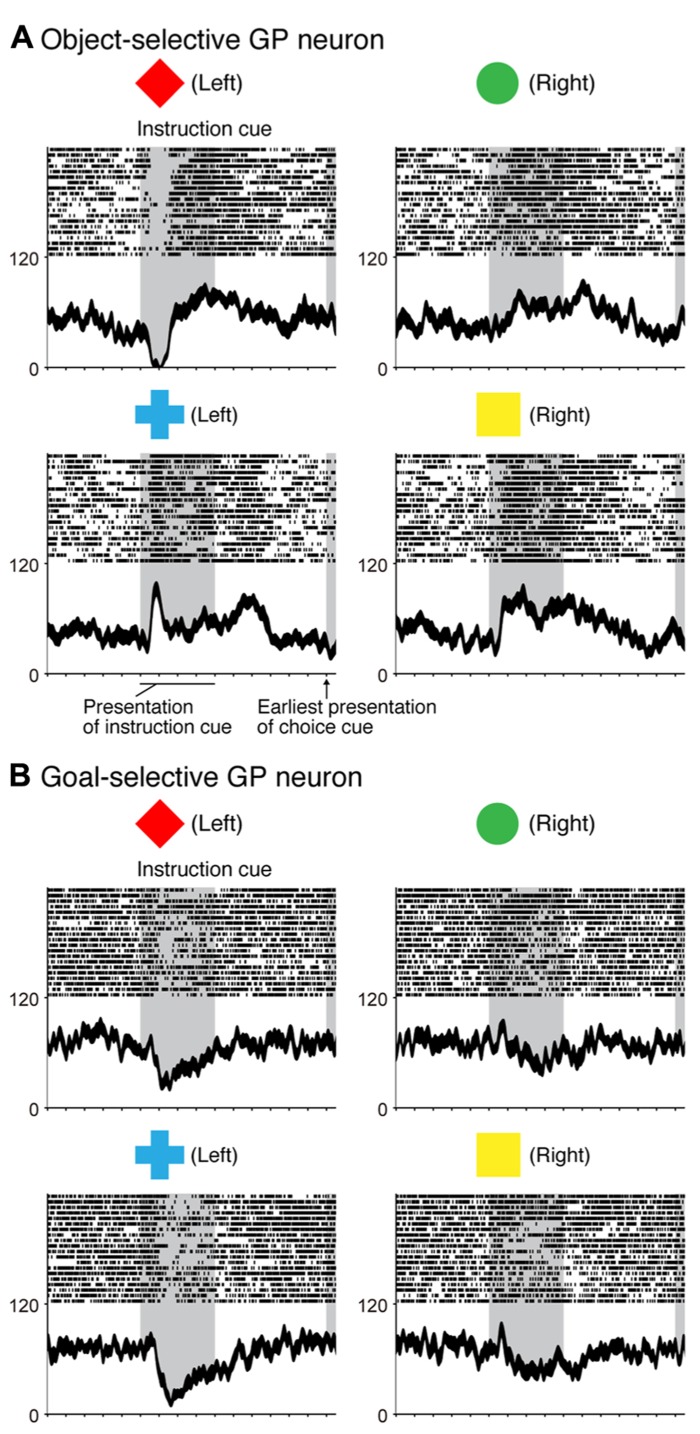
**Two examples of neurons in the GP selective for a visual object and a behavioral goal in the symbolic cue task. (A)** Activity of this GP neuron decreased when a red diamond was presented as an instruction cue, whereas it increased when a blue cross was presented as an instruction cue. **(B)** Activity of this GP neuron decreased when either a red diamond or a blue cross was presented. Neuronal activity was aligned with the onset of the instruction cue. The gray areas on the left indicate when the instruction was presented, and the gray areas on the right represent the earliest presentation of the choice cue. The tick marks on the horizontal axis are placed at 200-ms intervals. The display formats are the same as those used in **Figure 7** (adapted from Arimura et al., 2013).

In the context of this evidence against the hierarchical organization of goal development, the areas from which the PMd receives goal signals remain unidentified. To address this issue, we examined neurons in the BG while monkeys performed the symbolic cue task. We recorded neurons in the GP of the BG while monkeys performed the task (Arimura et al., 2013). GP neurons were considered to carry signals within the BG at the output stage (the internal segment, GPi) and at the intermediate stage (the external segment, GPe) of a series of information-processing steps. Thus, comparing the neuronal response properties in the GP with those in the PMd and lateral PFC would lead to a better understanding of the involvement of cortico-BG circuits in conditional visuo-goal association. When the instruction cue appeared, a subset of GP neurons started to reflect visual features (**Figure 12A**), and selectivity developed as early as it did in vlPFC neurons (**Figure 11A**). This prompt representation of visual objects by BG neurons is consistent with previous reports (Caan et al., 1984; Brown et al., 1995; Yamamoto et al., 2012, 2013; Yasuda et al., 2012). Subsequently, GP neurons began to reflect goals that were informed by the visual signals (**Figure 12B**), and the timing of selectivity development was no later than it was in the PMd, vlPFC, and dlPFC (**Figure 11B**). These observations indicate that the GP is involved in the early determination of behavioral goals, suggesting that the GP may emit a signal to inform wide cortical areas that a certain object or goal has appeared, serving to trigger subsequent information processing in these areas. The representation of an abstract aspect of motor behavior is consistent with prior reports showing that neurons in the putamen and GP represent a target position or a movement direction as the intended movement direction (Mitchell et al., 1987; Alexander and Crutcher, 1990b, 1990c). Clinical studies have reported that BG dysfunction results in deficits in cognitive processes (Mendez et al., 1989; Dubois and Pillon, 1997; Crucian and Okun, 2003; Uc et al., 2005). The loss of neurons representing abstract aspects of behavior may underlie these deficits.

## INVOLVEMENT OF CORTICO-BG CIRCUITS IN SELECTION OF ACTION BASED ON A GOAL

Monkeys participating in the symbolic cue task could specify or select the forthcoming action (the absolute position of a target on the screen) after the appearance of the choice cue. We found that neuronal activity selective for actions developed in the GP as well as in the PMd, dlPFC, and vlPFC. In contrast to the timing of the development of goal selectivity, the timing of the development of action selectivity in the GP differed from that in cortical areas; action representation in the GP emerged 30 ms later than it did in the cortical areas (**Figure 11C**). Furthermore, neurons that integrated representations of goals with choice-cue locations, which are considered to play a crucial role in the transformation from goal to action, were less numerous in the GP than in the PMd (**Figure 8B**). Muhammad et al. (2006) reported that behavioral responses in a visuomotor task employing the GO/NO-GO paradigm tended to begin earlier in the PMd than in the striatum. Antzoulatos and Miller (2011) showed that the lateral PFC plays a major role in the abstract categorization of visual signals for executing saccadic eye movements. Seo et al. (2012) revealed that representation of a selected action occurred earlier in the lateral PFC than in the dorsal striatum. Taken together, these data suggest that an action command determination based on visual signals is initially specified in cortical areas such as the PMd and lateral PFC, and this is followed by representation in the GP. This suggests that the BG do not play a major role in the process by which a behavioral goal is transformed into an action or in specifying an action based on a goal. Rather, the BG may be involved in registering an established action, based on which, competing motor programs are suppressed (Mink, 1996) or subsequent processes for action preparation and execution are initiated.

## NEURAL COMPUTATIONS OF CORTICO-BG CIRCUITS

**FIGURE 13:**
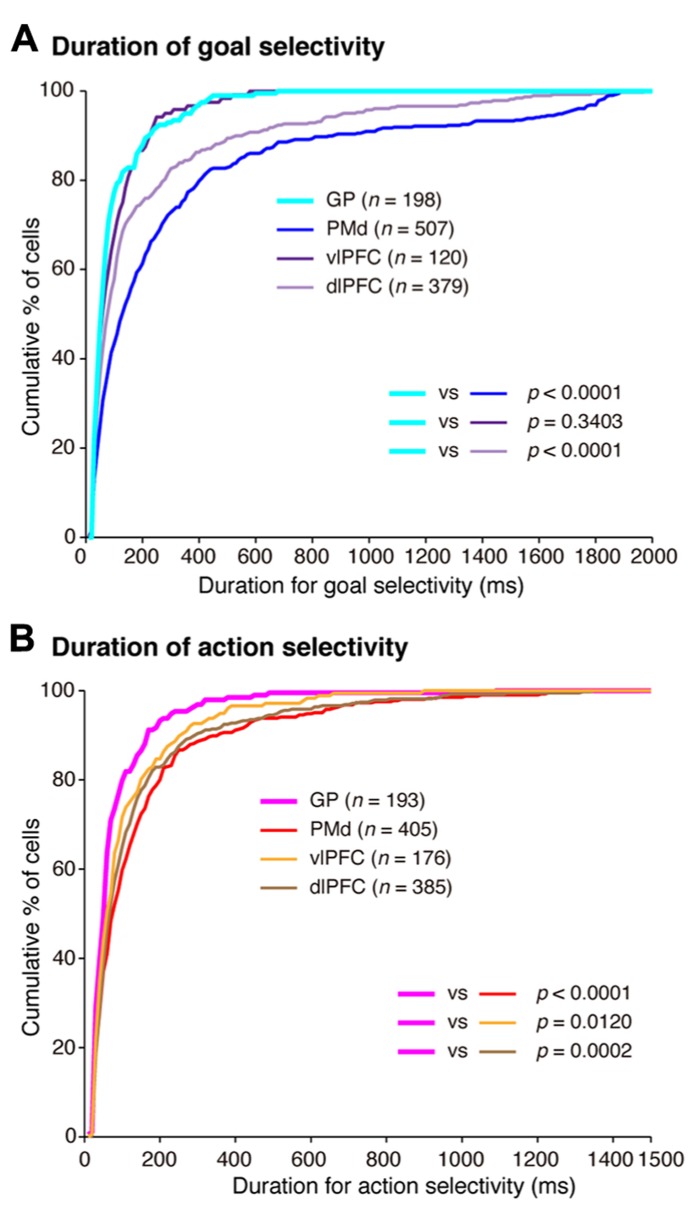
**Temporal profiles of neuronal selectivity for behavioral goals and actions in the symbolic cue task. (A)** Cumulative fractions of the duration of goal selectivity in the GP (light blue), PMd (dark blue), vlPFC (dark purple), and dlPFC (light purple) after instruction cue-onset in the symbolic cue task. **(B)** Cumulative fractions of the duration of action selectivity in the GP (pink), PMd (red), vlPFC (orange), and dlPFC (brown) after choice-cue presentation in the symbolic cue task. In **(A)** and** (B)**, the parentheses enclose the number of neurons with an onset of selectivity ≤2,000 ms after instruction-cue onset (for goal selectivity, **A**) and ≤1,500 ms after choice-cue onset (for action selectivity, **B**). The *p*-values indicate the results of the statistical analysis (Kolmogorov–Smirnov test) examining differences between neurons in the GP and the three cortical areas (adapted from Arimura et al., 2013).

In a series of studies on conditional visuo-goal association (Nakayama et al., 2008; Yamagata et al., 2009, 2012; Arimura et al., 2013), neurons from both the cortical areas (the PMd, vlPFC, and dlPFC) and the BG (GP) were recorded. This provided an opportunity to analyze activity with the aim of gaining insights into the neural computations of cortico-BG circuits. According to Marsden (1982), “the BG might focus attention on a single event in the environment to the exclusion of all others” (p. 512). Additionally, Houk and Wise (1995) proposed that the BG may play a role in contextual pattern recognition. According to this theory, GP neurons transiently decrease or increase activity, giving rise to sustained activity enhancement (context registration) or suppression (context negation) in the thalamus and cerebral cortex. Graybiel (2008) revealed that the BG are involved in representing behavioral boundaries. Consistent with these, we observed that goal and action representations by each GP neuron were transient in nature and much briefer than were those in the PMd and dlPFC (**Figure 13**). In contrast, the duration of goal selectivity of vlPFC neurons was comparable to that of GP neurons, supporting the hypothesis that vlPFC is not essential for maintaining working memory (Rushworth et al., 1997). Although the duration of the goal and action representations of GP neurons was shorter, the magnitude of the selective responses representing the goal and action were considerable: the mean activity modulation of GP neurons amounted to 16–47 spikes/s. The potency of neuronal responses was further characterized by the promptness of activity modulation, which was revealed by population selectivity, as selectivity peaked shortly (<400 ms) after the onset of the instruction and choice cues. Overall, the GP codes information via highly active neurons with short-lasting selectivity. This type of information coding is known as sparse coding and is thought to constitute a critical mechanism underpinning sensory (Olshausen and Field, 2004) and motor processing (Hahnloser et al., 2002). Taken together, our data suggest that the BG may employ sparse coding in the determination of behavioral goals and the specification of actions, whereas the PMd and dlPFC neurons are involved in maintaining the determined goals and specified actions with sustained responses, as well as in goal determination and action specification.

## FUTURE DIRECTIONS

The data and hypotheses discussed in the present study should be expanded in several directions to gain deeper insights into the neural mechanisms underlying conditional visuo-goal association. First, although we focused on the lateral frontal cortex, other cortical areas may also play a role, including the orbitofrontal cortex, the anterior cingulate cortex, the frontal polar cortex, the pre-SMA and the posterior parietal cortex (Matsuzaka et al., 1992; Matsuzaka and Tanji, 1996; Sakai et al., 1999; Hernández et al., 2002; Hoshi and Tanji, 2004b; Stoet and Snyder, 2004; Diedrichsen et al., 2006; Freedman and Assad, 2006; Kamigaki et al., 2009; Tsujimoto et al., 2009, 2010, 2011; Amiez et al., 2012; Luk and Wallis, 2013). Because these areas are interconnected with the networks involving the PMd, lateral PFC, and BG (Selemon and Goldman-Rakic, 1985; Barbas and Pandya, 1989; Luppino et al., 1993; Matelli et al., 1998; Petrides and Pandya, 1999, 2002; Ongur and Price, 2000; Haber et al., 2006; Rozzi et al., 2006; Morecraft et al., 2012; Haynes and Haber, 2013), it is suggested that a large-scale network underlies the goal-directed behavior mediated by conditional visuo-goal association. Second, we here focused on neural representations when the animals were familiar with the association between the visual stimuli and goals. However, it is also necessary to examine the mechanisms at different stages of association or rule learning because each area/network can play a specific role depending on these parameters (Schultz et al., 1997; Hikosaka et al., 1999; Doya, 2000; Samejima et al., 2005; Lee et al., 2012). For example, Amiez et al. (2012) showed in humans that as the learning of conditional visuomotor associations progresses, the areas active in relation to motor selection move from the cognitive networks involving the dlPFC, the caudate nucleus, and the PMd to the motor networks, including the putamen and the PMd. They also showed that that the orbitofrontal cortex and anterior cingulate cortex are active in relation to the evaluation of the consequences of a selected action. Consistent with this, neurons in the orbitofrontal cortex can represent response choices when feedback is provided (Tsujimoto et al., 2009, 2010), and neurons in the anterior cingulate cortex can interactively represent actions and rewards (Matsumoto et al., 2003). Third, we discussed the representation of goals in the spatial domain (spatial-specific goals; i.e., right vs. left). In future studies, goal representations in other domains should be examined. For example, neurons in the prefrontal cortex and the GP can encode object-specific goals, such as shape or color (Hoshi et al., 1998; Genovesio et al., 2012; Saga et al., 2013). Neural mechanisms for making associations between visual objects were identified in the prefrontal cortex (Hasegawa et al., 1998; Rainer et al., 1999) and the inferotemporal cortex (Sakai and Miyashita, 1991; Naya et al., 2001; Hirabayashi et al., 2013a, b). However, it remains unclear whether the same brain areas responsible for motor behavior based on spatial-specific goals support motor behavior based on object-specific goals.

## SUMMARY AND CONCLUSION

Previous studies based on a framework derived from conditional visuomotor association (**Figure 1A**) revealed neural mechanisms underlying the specification and planning of actions based on sensory signals. However, applications resting solely on this conceptualization encounter problems related to generalization and flexibility, which are essential processes in executive function. To overcome this problem, we extended this conceptualization and postulated a more general framework, conditional visuo-goal association (**Figure 1B**), in which the visual signal identifies an abstract behavioral goal, and an action is subsequently selected and executed to meet this goal. Neuronal activity recorded from the brain areas of monkeys performing a task involving conditional visuo-goal association revealed that they regulate the task in an area-dependent manner. By comparing the response properties of neurons in the GP, PMd, dlPFC, and vlPFC of monkeys engaging in goal-directed behavior mediated by conditional visuo-goal association, we revealed that these areas are commonly involved in the initial stages of goal determination based on visual signals. Neurons representing an abstract behavioral goal are considered to provide a foundation for executive function. In contrast, we found that GP activity follows the leading activity in the PMd, dlPFC, and vlPFC in specifying an action based on an abstract behavioral goal. Taken together with the finding that a shorter length of time represented goal and action by neurons in the GP compared with neurons in the PMd and dlPFC, these data suggest a unique involvement of the BG and the frontal cortical areas in goal-directed behavior. Increased understanding of the neural mechanisms underlying conditional visuo-goal association will yield deeper insights into the fundamental principles underpinning goal-directed behavior.

## Conflict of Interest Statement

The author declares that the research was conducted in the absence of any commercial or financial relationships that could be construed as a potential conflict of interest.
